# An Updated Review on Pharmaceutical Properties of Gamma-Aminobutyric Acid

**DOI:** 10.3390/molecules24152678

**Published:** 2019-07-24

**Authors:** Dai-Hung Ngo, Thanh Sang Vo

**Affiliations:** 1 Faculty of Natural Sciences, Thu Dau Mot University, Thu Dau Mot City 820000, Vietnam; 2 NTT Hi-Tech Institute, Nguyen Tat Thanh University, Ho Chi Minh City 700000, Vietnam

**Keywords:** anti-hypertension, bioactivity, Gaba, Gaba-rich product, health benefit

## Abstract

Gamma-aminobutyric acid (Gaba) is a non-proteinogenic amino acid that is widely present in microorganisms, plants, and vertebrates. So far, Gaba is well known as a main inhibitory neurotransmitter in the central nervous system. Its physiological roles are related to the modulation of synaptic transmission, the promotion of neuronal development and relaxation, and the prevention of sleeplessness and depression. Besides, various pharmaceutical properties of Gaba on non-neuronal peripheral tissues and organs were also reported due to anti-hypertension, anti-diabetes, anti-cancer, antioxidant, anti-inflammation, anti-microbial, anti-allergy, hepato-protection, reno-protection, and intestinal protection. Therefore, Gaba may be considered as potential alternative therapeutics for prevention and treatment of various diseases. Accordingly, this updated review was mainly focused to describe the pharmaceutical properties of Gaba as well as emphasize its important role regarding human health.

## 1. Introduction

Gamma-aminobutyric acid (Gaba) is a non-protein amino acid that is widely distributed in nature. Especially, Gaba is present in high concentrations in different brain regions [1]. Besides, it was also found in various foods such as green tea, soybean, germinated brown rice, kimchi, cabbage pickles, yogurt, etc. Generally, Gaba was produced by l-glutamic acid under the catalyzation of glutamic acid decarboxylase [2]. In the nervous system, newly synthesized Gaba is packaged into synaptic vesicles and then released into the synaptic cleft to diffuse to the target receptors on the postsynaptic surface [3]. Numerous studies have identified two distinct classes of Gaba receptor including Gaba_A_ and Gaba_B_ [4]. These receptors are different due to their pharmacological, electrophysiological, and biochemical properties. Gaba_A_ receptor is Gaba-gated chloride channels located on the postsynaptic membrane, while Gaba_B_ receptor is G protein-coupled receptors located both pre- and postsynaptic.

Gaba is well known as the major inhibitory neurotransmitter in the mammalian central nervous system. It was reported to play vital roles in modulating synaptic transmission, promoting neuronal development and relaxation, and preventing sleeplessness and depression [5,6,7,8,9]. Notably, various biological activities of Gaba were documented due to anti-hypertension, anti-diabetes, anti-cancer, antioxidant, anti-inflammation, anti-microbial, and anti-allergy. Moreover, Gaba was also reported as a protective agent of liver, kidney, and intestine against toxin-induced damages [10]. In this contribution, the pharmaceutical properties of Gaba on non-neuronal peripheral tissues and organs were mainly focused to emphasize its beneficial role in prevention and treatment of various diseases.

## 2. Pharmaceutical Properties of Gaba

### 2.1. Neuroprotective Effect

**Figure 1:**
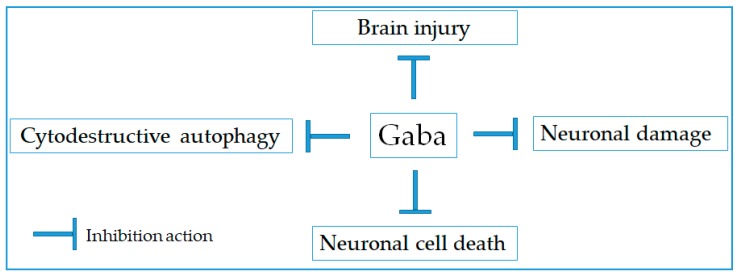
Therapeutic targets for neuroprotective activity of Gaba.

It has been reported that the damage of nervous tissue triggers inflammatory response, causing the release of various inflammatory mediators such as reactive oxygen species (ROS), nitric oxide, and cytokines. These mediators can cause several neuronal degenerations in the central nervous system such as Alzheimer’s, Parkinson’s, and multiple sclerosis [11,12]. So far, numerous studies have been reported regarding the important roles of Gaba on neuro-protection against the degeneration induced by toxin or injury (Figure 1 and Table 1). According to Cho et al. (2007), Gaba produced by the kimchi-derived *Lactobacillus buchneri* exhibited a protective effect against neurotoxic-induced cell death [13]. Moreover, Gaba-enriched chickpea milk can protect neuroendocrine PC-12 cells from MnCl_2_-induced injury, improve cell viability, and reduce lactate dehydrogenase release [14]. On the other hand, Zhou and colleagues have determined that Gaba receptor agonists also possessed neuroprotective effect against brain ischemic injury. Both Gaba_A_ and Gaba_B_ receptor agonist (muscimol and baclofen) could significantly protect neurons from the death induced by ischemia through increasing nNOS (Ser847) phosphorylation [15]. Likewise, the administration of Gaba_B_ receptor agonist baclofen significantly alleviated neuronal damage and suppressed cytodestructive autophagy via up-regulating the ratio of Bcl-2/Bax and increasing the activation of Akt, GSK-3β, and ERK [16]. Additionally, co-activation of Gaba receptor agonists (muscimol and baclofen) resulted in the attenuation of Fas/FasL apoptotic signaling pathway, inhibition of the kainic acid-induced increase of thioredoxin reductase activity, the suppression of procaspase-3 activation, and the decrease in caspase-3 cleavage. It indicates that co-activation of Gaba receptor agonists results in neuroprotection by preventing caspase-3 denitrosylation in kainic acid-induced seizure of rats [17].

### 2.2. Neurological Disorder Prevention

**Figure 2:**
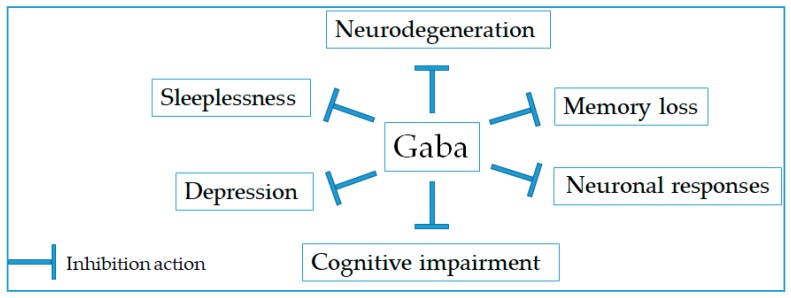
Preventive action of Gaba on neurological disorders.

Neurologic disorder is associated to dysfunction in part of the brain or nervous system, resulting in physical or psychological symptoms. It includes epilepsy, Alzheimer’s disease, cerebrovascular diseases, multiple sclerosis, Parkinson’s disease, neuroinfections, and insomnia [18]. It was evidenced that Gaba can suppress neurodegeneration and improve memory as well as cognitive functions of the brain (Figure 2 and Table 2). According to Okada et al. (2000), the usefulness of Gaba-enriched rice germ on sleeplessness, depression, and autonomic disorder was examined [19]. Twenty female patients were administered by Gaba-rich rice germ for three times per day. It was observed that the most common mental symptoms during the menopausal and pre-senile period such as sleeplessness, somnipathy, and depression were remarkedly improved in more than 65% of the patients with such symptoms. Likewise, oral administration of Gaba-rich Monascus-fermented product exhibited the protective effect against depression in the forced swimming rat model. Its antidepressant effect was suggested due to recovering the level of monoamines norepinephrine, dopamine, and 5-hydroxytryptamine in the hippocampus [20]. Meanwhile, Yamatsu et al. (2016) reported that Gaba administration significantly shortened sleep latency and increased the total non-rapid eye movement sleep time, indicating the essential role of Gaba in the prevention of a sleep disorder [21]. Moreover, the mixture of Gaba and l-theanine could decrease sleep latency, increase sleep duration, and up-regulate the expression of Gaba and glutamate GluN1 receptor subunit [22]. On the other hand, the electroencephalogram assay has revealed the significantly roles of Gaba in increasing alpha waves, decreasing beta waves, and enhancing IgA levels under stressful conditions. It indicates that Gaba is able to induce relaxation, diminish anxiety, and enhance immunity under stressful conditions [23]. The administration of Gaba-enriched product fermented by kimchi-derived lactic acid bacteria also improved long-term memory loss recovery in the cognitive function-decreased mice and increased the proliferation of neuroendocrine PC-12 cells in vitro [24]. Moreover, the Gaba-enriched fermented *Laminaria japonica* (GFL) provided a protective effect against cognitive impairment associated with dementia in the elderly [25]. In addition, Reid and colleagues have shown that GFL could improve cognitive impairment and neuroplasticity in scopolamine- and ethanol-induced dementia model mice [26]. Especially, GFL was effective in increasing serum brain-derived neurotrophic factor level that associated with lower risk for dementia and Alzheimer’s disease in middle-aged women [27]. These results indicate that the use of Gaba-enriched functional foods may improve depression, sleeplessness, cognitive impairment, and memory loss.

### 2.3. Anti-Hypertensive Effect

Hypertension is known to relate to a high blood pressure condition, causing various cardiovascular diseases such as ischemic and hemorrhagic stroke, myocardial infarction, and heart and kidney failure [28]. Particularly, angiotensin-I converting enzyme (ACE) was revealed to play an important role in the regulation of blood pressure via converting angiotensin I into the potent vasoconstrictor angiotensin II [29]. Hence, ACE is one of the among therapeutic targets for the control of hypertension. According to Nejati et al. [30], the milk fermented by *Lactococcus lactis* DIBCA2 and *Lactobacillus plantarum* PU11 exhibited an ACE inhibitory activity up to an IC_50_ value of 0.70 ± 0.07 mg/mL. Similarly, high ACE inhibitory activity was also observed by Gaba, which was achieved from *L. plantarum* NTU 102-fermented milk [31]. Moreover, *L. brevis*-fermented soybean containing approximately 1.9 g/kg Gaba was found to possess higher ACE inhibitory activity than the traditional soybean product [32]. Besides, the fermentation of a soybean solution by kimchi-derived lactic acid bacteria in the optimized condition has achieved a Gaba content of up to 1.3 mg/g soybean seeds, and its ACE inhibitory activity was observed up to 43% as compared to the control [33]. Notably, high Gaba content (10.42 mg/g extract) and significant ACE inhibitory activity (92% inhibition) was also determined by the fermented lentils [34].

On the other hand, the anti-hypertensive activity of Gaba was also reported in numerous studies using different experimental models (Table 3). Kimura et al. [35] have investigated the effect of Gaba on blood pressure in spontaneously hypertensive rats. It was observed that the intraduodenal administration of Gaba (0.3 to 300 mg/kg) caused a dose-related decrease in the blood pressure in 30 to 50 min. The hypotensive effect of Gaba was suggested due to attenuating a sympathetic transmission through the activation of the Gaba_B_ receptor at presynaptic or ganglionic sites. Moreover, the lowering effect of Gaba-enriched dairy product on the blood pressure of spontaneously hypertensive and normotensive Wistar-Kyoto rats was also determined [36]. Notably, the clinical trial has confirmed that daily supplementation of 80 mg of Gaba was effective in the reduction of blood pressure in adults with mild hypertension [37]. Therefore, the consumption of Gaba-enriched dairy product would be beneficial for the down-regulation of hypertension. Indeed, the administration of Gaba-enriched rice grains brings about 20 mmHg decrease in blood pressure in spontaneously hypertensive rats, while there was no significant hypotensive effect in normotensive rats [38]. Likewise, the significant anti-hypertensive activity and the serum cholesterol-lowering effect of Gaba-rich brown rice were shown in spontaneously hypertensive rats as compared to the control [39,40]. In the clinical trial, the effects of Gaba-enriched white rice on blood pressure in 39 mildly hypertensive adults has been examined in a randomized, double blind, placebo-controlled study [41]. It was revealed that the consumption of the Gaba rice could improve the morning blood pressure as compared with the placebo rice after the 1st week and during the 6th and 8th weeks. In the same trend, Tsai and colleagues have determined that Gaba-enriched Chingshey purple sweet potato-fermented milk by lactic acid bacteria (*L. acidophilus* BCRC 14065, *L. delbrueckii* ssp. lactis BCRC 12256, and *L. gasseri* BCRC 14619) was able to reduce both systolic blood pressure and diastolic blood pressure in spontaneously hypertensive rats [42]. The alleviative effect of probiotic-fermented purple sweet potato yogurt on cardiac hypertrophy in spontaneously hypertensive rat hearts was also further determined by Lin and colleagues [43].

In addition, the other Gaba-rich products from bean, tomato, and bread were also reported to be effective in the attenuation of hypertension in vivo. Definite decreases in systolic and diastolic blood pressure values and blood urea nitrogen level were achieved in spontaneously hypertensive rats fed with Gaba-enriched beans [44,45]. Likewise, the anti-hypertensive activity of a Gaba-rich tomato was evidenced to decrease blood pressure in spontaneously hypertensive rats significantly [46]. Moreover, the blood pressure of patients with pre- or mild- to moderate hypertension was significantly decreased during the consumption of 120 g/day of Gaba-rich bread [47]. Accordingly, Gaba-enriched dairy foods may be preferred to use for anti-hypertensive therapeutics.

### 2.4. Anti-Diabetic Effect

**Figure 3:**
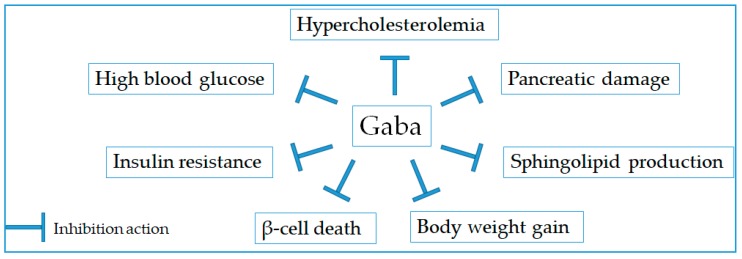
Therapeutic targets for anti-diabetic activity of Gaba.

Diabetes is an endocrine disorder that is associated with dysregulation of carbohydrate metabolism and deficiency of insulin secretion or insulin action, causing chronic hyperglycemia [48]. So far, diabetic diseases can be managed by pharmacologic interventions [49]. However, the lowering blood glucose effect of pharmacological drugs is accompanied with various disadvantages such as drug resistance, side effects, and even toxicity [50]. Therefore, the proper diet and exercise have been recommended and preferred as alternative therapeutics for the regulation of diabetic diseases. Notably, Gaba and Gaba-enriched natural products have been evidenced as effective agents in lowering blood glucose, attenuating insulin resistance, stimulating insulin release, and preventing pancreatic damage (Figure 3 and Table 4). Soltani and colleagues have shown that Gaba enhanced islet cell function via producing membrane depolarization and Ca^(2+)^ influx, activating PI3-K/Akt-dependent growth and survival pathways, and restoring the β-cell mass [51]. Moreover, Gaba preferentially up-regulated pathways linked to β-cell proliferation and rose to a distinct subpopulation of β cells with a unique transcriptional signature, including urocortin3, wnt4, and hepacam2 [52]. Especially, the combined use of Gaba and sitagliptin was superior in increasing β-cell proliferation, reducing cell apoptosis, and suppressing α-cell mass [53]. On the other hand, Gaba was found to enhance insulin secretion in pancreatic INS-1 β-cells [54]. In the pre-clinical trial model, Gaba administration could decrease the ambient blood glucose level and improve the glucose excursion rate in streptozotocin-induced diabetic mice [53]. Furthermore, oral treatment with Gaba significantly reduced the concentrations of fasting blood glucose, improved glucose tolerance and insulin sensitivity, and inhibited the body weight gain in the high fat diet-fed mice [55]. Notably, Gaba potentially inhibited the diabetic complication related to the nervous system via suppressing the Fas-dependent and mitochondrial-dependent apoptotic pathway in the cerebral cortex [56].

The fact that the germination of rice and the fermentation of foods are accompanied with the increase in Gaba content [57,58], therefore, the pre- and germinated rice and fermented foods were highly appreciated for their roles in positive regulation of diabetes and its complication. According to Hagiwara and colleagues, the feeding of pre-germinated brown rice diet to diabetic rats significantly decreased blood glucose, adipocytokine PAI-1 concentration, and plasma lipid peroxide [59]. Moreover, pre-germinated brown rice lowered HbA(1c) and adipocytokine (TNF-α and PAI-1) concentration and increased the adiponectin level in type-2 diabetic rats, leading to the prevention of potential diabetic complications [60]. In addition, high fat diet-induced diabetic pregnant rats fed with the germinated brown rice lead to the increase in adiponectin levels and the reduction of insulin, homeostasis model assessment of insulin resistance, leptin, and oxidative stress in their offspring [61]. On the other hand, blackish purple pigmented rice with a giant embryo significantly decreased blood glucose and plasma insulin levels, adipokine concentrations, and hepatic glucose-regulating enzyme activities in ovariectomized rats [62]. Meanwhile, glucose homeostasis was greatly improved through the intervention of Gaba-enriched wheat bran in the context of a high-fat diet rat [63]. The supplement of Gaba-enriched rice bran to obese rats also exhibited an efficient effect on lowering serum sphingolipids, a marker of insulin resistance [64]. In clinical trials, Ito and colleagues have suggested that the intake of pre-germinated brown rice was effective in lowering postprandial blood glucose concentration without insulin secretion increase [65]. Likewise, Hsu et al. [66] and Suzuki et al. [67] have confirmed that pre-germinated brown rice decreased blood glucose and hypercholesterolemia in type 2 diabetes patients.

Beside germinated rice, fermented foods are also known to contain a significant amount of Gaba and possess potential anti-diabetic activity. The oral administration of hot water extract of the fermented tea obtained by tea-rolling processing of loquat (*Eriobotrya japonica*) significantly decreased the blood glucose level and serum insulin secretion in maltose-loaded Sprague–Dawley rats [68]. Similarly, anti-diabetic effects of green tea fermented by cheonggukjang was observed via decreasing water intake and lowering blood glucose and HbA1c levels in diabetic mice [69]. In addition, mung bean fermented by *Rhizopus* sp. [70], yogurt fermented by *Streptococcus salivarius* subsp. thermophiles fmb5 [71], and soybean extract fermented by *Bacillus subtilis* MORI [72] could enhance their anti-hyperglycemic effect via reducing blood glucose, HbA1c, cholesterol, triglyceride, and low-density lipoprotein levels in diabetic mice. In the same trend, the milk fermented by commercial strain YF-L812 (*S. thermophilus*, *L. delbrueckii* subsp. *bulgaricus*), standard strains. *B. breve* KCTC 3419, and *L. sakei* LJ011. Fermented milk was effective in decreasing fasting blood glucose, serum insulin, leptin, glucose and insulin tolerance, total cholesterol, triglycerides, and low density lipoprotein cholesterol [73]. Especially, the consumption of probiotic-fermented milk (kefir) by type 2 diabetic patents lowered HbA1C level, homeostatic model assessment of insulin resistance, and homocysteine amount [74,75]. Accordingly, the germinated rice and fermented foods, which contain a high amount of Gaba, could be used as anti-diabetic functional food for maintaining health and preventing complications in type 2 diabetes.

### 2.5. Anti-Cancer Effect

Cancer is involved in the unregulated cell proliferation, apoptosis suppression, invasion, and metastasis [76]. Current cancer therapies are related to surgery, radiation treatment, and chemotherapy treatment, which are widely applied for treatment of all kinds of cancers. However, these therapies possess major disadvantages including cancer recurrence, drug resistance, and side effects. Hence, the discovery of alternative medicines with desirable properties is always necessary. In this regard, Gaba was emerged as a promising compound that is able to regulate cancer due to the induction of apoptosis and inhibition of proliferation and metastasis (Table 5). Gaba-enriched brown rice extract significantly retarded the proliferation rates of L1210 and Molt4 leukemia cells and enhanced apoptosis of the cultured L1210 cells [77]. Moreover, Schuller et al. [78] suggested that Gaba had a tumor suppressor function in small airway epithelia and pulmonary adenocarcinoma, providing the approach for the prevention of pulmonary adenocarcinoma in smokers. According to Huang and colleagues, Gaba was determined to inhibit the activity and expression of MMP-2 and MMP-9 in cholangiocarcinoma QBC939 cells, suggesting its role in prevention of invasion and metastasis in cancer [79]. Song and colleagues also found the inhibitory effects of Gaba on the proliferation and metastasis of colon cancer cells (SW480 and SW620 cells) due to the up-pressing cell cycle progression (G2/M or G1/S phase), attenuating mRNA expression of EGR1-NR4A1 and EGR1-Fos axis, and disrupting MEK-EGR1 signaling pathway [80]. Especially, the co-treatment of Gaba and Celecoxib significantly inhibited systemic and tumor VEGF, PGE_2_, and cAMP molecules and down-regulated COX-2 and p-5-LOX protein in pancreatic cancer cells [81]. Moreover, the prolonged administration of Gaba at 1000 mg/kg body weight significantly decreased the number of gastric cancers of the glandular stomach in Wk 52 rats. In parallel, the histological method also revealed the role of Gaba on decreasing the labeling index of the antral mucosa and increasing the serum gastrin level [82]. Likewise, the pre-treatment of Gaba also significantly reduced intrahepatic liver metastasis and primary tumor formation in mice and inhibited human liver cancer cell migration and invasion via the induction of liver cancer cell cytoskeletal reorganization [83]. Meanwhile, the increase in the activity of Gaba_A_ receptor contributed to the down-regulation of alpha-fetoprotein mRNA expression and cell proliferation in malignant hepatocyte cell line [84].

### 2.6. Antioxidant Effect

**Figure 4:**
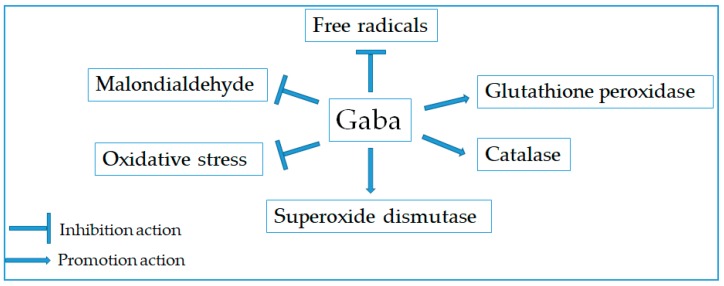
Modulatory activity of Gaba for antioxidant promotion.

The free radicals contain one or more unpaired electrons that are generated from the living organisms and external sources. The high level of free radicals could cause the damage of the body’s tissues and cells, leading to human aging and various diseases [85,86]. Thus, consumption of natural products with high anti-oxidant effect is useful for the prevention of free radical-caused diseases [86]. Herein, the antioxidant property of Gaba has been evidenced in numerous studies (Figure 4). It was shown that Gaba was able to trap the reactive intermediates during lipid peroxidation and react readily with malondialdehyde under physiological conditions [87]. Moreover, the administration of Gaba significantly decreased malondialdehyde concentration and increased the activity of superoxide dismutase and glutathione peroxidase in the cerebral cortex and hippocampus of acute epileptic state rats [88]. In other studies, the protective effect of Gaba against H_2_O_2_-induced oxidative stress in pancreatic cells [89] and human umbilical vein endothelial cells [90] was observed via reducing cell death, inhibiting reactive oxygen species (ROS) production, and enhancing antioxidant defense systems. Similarly, gamma rays-induced oxidative stress in the small intestine of rats was significantly ameliorated via decreasing malondialdehyde and advanced oxidation protein productions, increasing catalase and glutathione peroxidase activities, preventing mucosal damage and hemorrhage, and inducing the regeneration of the small intestinal cells [91]. Gaba also attenuated brain oxidative damage associated with insulin alteration in streptozotocin-treated rats [92]. On the other hand, Gaba from *L. brevis*-fermented sea tangle solution was observed to exhibit stronger antioxidant activity than positive control BHA in scavenging DPPH and superoxide radicals and inhibiting xanthine oxidase [93]. Meanwhile, the Gaba-rich germinated brown rice extract considerably scavenged hydroxyl radical and thiobarbituric acid-reactive substances in both cell-free medium and post-treatment culture media, indicating its radical scavenging capacity in both direct and indirect action [94]. Recently, brew-germinated pigmented rice vinegar was also suggested as a new product with high antioxidant activity [95].

### 2.7. Anti-Inflammatory Effect

**Figure 5:**
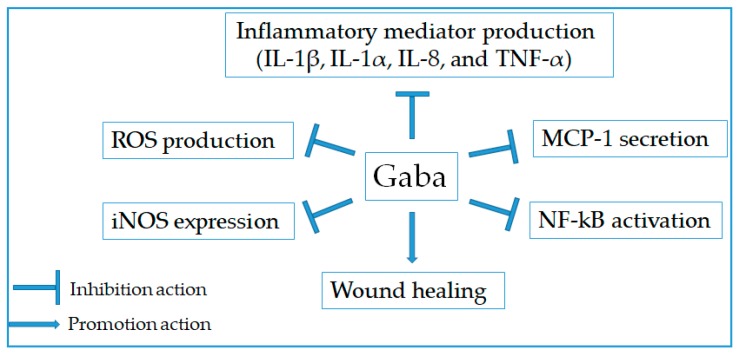
Therapeutic targets for anti-inflammatory activity of Gaba.

Inflammation response is triggered by the stimulation of various factors such as physical damage, ultra violet irradiation, microbial invasion, and immune reactions [96]. It is associated with the production of a large range of pro-inflammatory mediators such cytokine, NO, and PGE_2_ [97]. Notably, Gaba was indicated as an inhibitor of inflammation via decreasing pro-inflammatory mediator production and ameliorating inflammatory symptom (Figure 5). At the early time, Han et al. [98] have determined the anti-inflammatory activity of Gaba via inhibiting the production and expression of iNOS, IL-1β, and TNF-α in LPS-stimulated RAW 264.7 cells. As the result, it contributed to the reduction of the whole healing period and enhancement of wound healing at the early stage. Likewise, Gaba suppressed inflammatory cytokine production and NF-kB inhibition in both lymphocytes and pancreatic islet beta cells [99]. Recently, Gaba-enriched sea tangle *L. japonica*, Gaba-rich germinated brown rice, and Gaba-rich red microalgae *Rhodosorus marinus* were reported for their inhibitory capacities on inflammatory response. Gaba-enriched sea tangle *L. japonica* extract suppressed nitric oxide production and inducible nitric oxide synthase expression in LPS-induced mouse macrophage RAW 264.7 cells [100]. Gab-rich germinated brown rice inhibited IL-8 and MCP-1 secretion and ROS production from Caco-2 human intestinal cells activated by H_2_O_2_ and IL-1β [101]. Gaba-rich red microalgae *Rhodosorus marinus* extract negatively modulated expression and release of pro-inflammatory IL-1α in phorbol myristate acetate-stimulated normal human keratinocytes, therefore indicating the potential treatment of sensitive skins, atopia, and dermatitis [102]. Besides, the roles of Gaba in the attenuation of gut inflammation and improvement of gut epithelial barrier were suggested via inhibiting IL-8 production and stimulating the expression of tight junction proteins as well as the expression of TGF-β cytokine in Caco-2 cells [103].

### 2.8. Anti-Microbial Effect

Gaba tea is a kind of Gaba-enriched tea by the repeating treatments of alternative anaerobic and aerobic conditions. The Gaba tea extract exhibited inhibitory activity against *Vibrio parahaemolyticus, Staphylococcus aureus, Bacillus cereus, Salmonella typhimurium,* and *Escherichia coli* [104]. Gaba could increase *Pseudomonas aeruginosa* virulence due to stimulation of cyanogenesis, reduction in oxygen accessibility, and overexpression of oxygen-scavenging proteins. Gaba also promotes specific changes in the expression of thermostable and unstable elongation factors involved in the interaction of the bacterium with the host proteins [105]. Recently, the role of Gaba in anti-microbial host defenses was elucidated by Kim and colleagues [106]. Treatment of macrophages with Gaba enhanced phagosomal maturation and anti-microbial responses against mycobacterial infection. This study identified the role of Gabaergic signaling in linking anti-bacterial autophagy to enhance host innate defense against intracellular bacterial infection including *Mycobacteria, Salmonella,* and *Listeria*.

### 2.9. Anti-Allergic Effect

**Figure 6:**
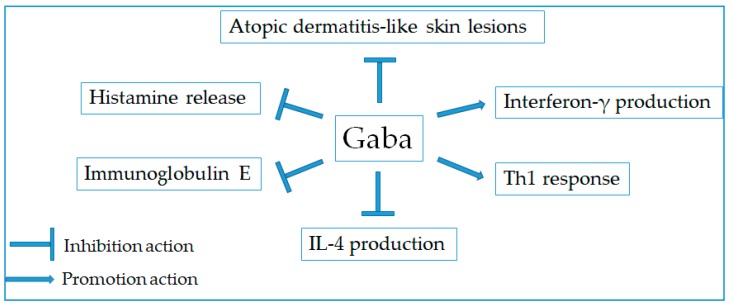
Therapeutic targets for anti-allergic activity of Gaba.

Allergy is a disorder of the immune system associating with an exaggerated reaction of the immune system to harmless environmental substances. Allergic reaction is characterized by the excessive activation of mast cells and basophils, leading to release various mediators such as histamine and an array of cytokines [107]. Among them, histamine is considered as the major target for potential anti-allergic therapeutics. Herein, the inhibitory activity of Gaba on histamine release from the activated mast cells was investigated in vitro [108,109]. Rat basophilic leukemia cells and rat peritoneal exudate cells sensitized with anti-dinitrophenyl (DNP) IgE and challenged with DNP-conjugated bovine serum albumin resulted in the release of histamine in a cell culture medium. However, IgE-mediated histamine release was inhibited by Gaba treatment in both cells. Conversely, the inhibitory activities of Gaba were lowered by the addition of CGP35348, a Gaba_B_ receptor antagonist. It indicated that Gaba inhibited degranulation from basophils and mast cells via Gaba_B_ receptor on the cell surface. On the other hand, Hokazono et al. [110] have evaluated the protective effect of Gaba against the development of atopic dermatitis (AD)-like skin lesions in NC/Nga mice. It was observed that Gaba could prevent the development of AD-like skin lesions in mice via alleviating serum immunoglobulin E (IgE) and splenocyte IL-4 production. The combined administration of Gaba and the fermented barley extract remarkedly increased splenic cell interferon-γ production, indicating the domination of Th1/Th2 balance to Th1 response. Hence, the simultaneous intake of Gaba and the fermented barley extract was encouraged to ameliorate allergic symptoms such as atopic dermatitis (Figure 6).

### 2.10. Hepatoprotective Effect

**Figure 7:**
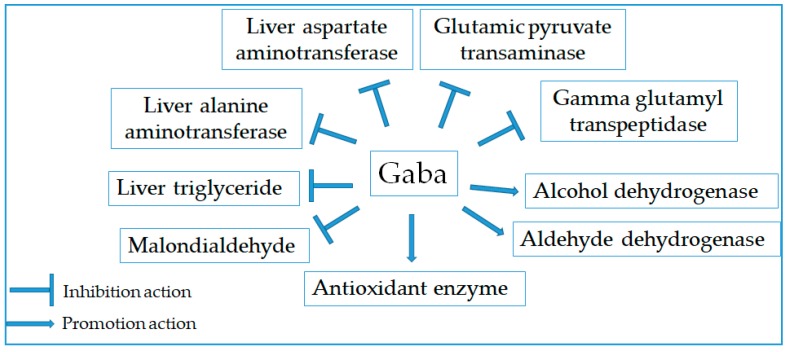
Mechanism of the action of Gaba for hepatoprotection.

The long-term use of ethanol can cause liver damage and unfavorable lipid profiles in humans. The toxic acetaldehyde is formed from alcohol under catalysis of alcohol dehydrogenase, causing various adverse effects such as thirst, vomiting, fatigue, headache, and abdominal pain [111]. For the first time, Oh and colleagues have evaluated the protective effect of Gaba-rich germinated brown rice against the toxic consequences of chronic ethanol use [112]. Interestingly, serum low-density lipoprotein cholesterol, liver aspartate aminotransferase, and liver alanine aminotransferase levels were decreased in mice fed both ethanol and brown rice extract for 30 days. Furthermore, the brown rice extract significantly increased serum and liver high-density lipoprotein cholesterol concentrations and reduced liver triglyceride and total cholesterol concentrations. In the same trend, Lee et al. [113] have reported that Gaba-rich fermented sea tangle (GFST) could prevent ethanol and carbon tetrachloride-induced hepatotoxicity in rats. The oral administration of GFST decreased the serum levels of glutamic pyruvate transaminase, gamma glutamyl transpeptidase, and malondialdehyde levels and increased antioxidant enzyme such as superoxide dismutase, catalase, and glutathione peroxidase [113]. Moreover, GFST increased the activities and transcript levels of major alcohol-metabolizing enzymes, such as alcohol dehydrogenase and aldehyde dehydrogenase, and reduced blood concentrations of alcohol and acetaldehyde [114]. In an in vitro study, the protective effects of GFST against alcohol hepatotoxicity in ethanol-exposed HepG_2_ cells were revealed by preventing intracellular glutathione depletion, decreasing gamma-glutamyl transpeptidase activity, and suppressing cytochrome P450 2E1 enzyme expression [115]. These results indicated that Gaba-rich foods might have a pharmaceutical role in the prevention of chronic alcohol-related diseases (Figure 7).

### 2.11. Renoprotective Effect

**Figure 8:**
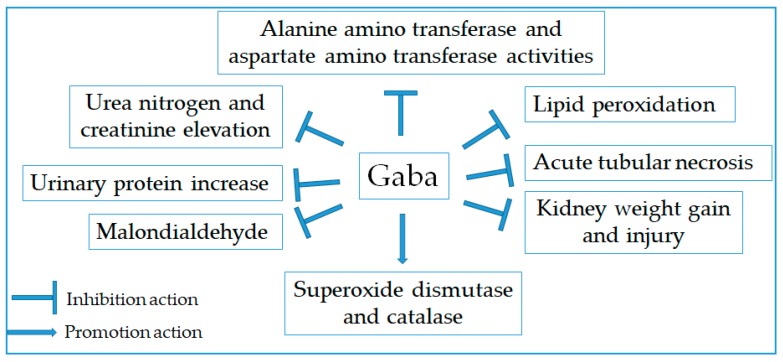
Mechanism of the action of Gaba for renoprotection.

Acute kidney injury is involved in kidney damage and cell death, causing high morbidity and mortality worldwide [116]. The renoprotective agents derived from natural products may be essential for the prevention or treatment of kidney injury-related diseases. Indeed, numerous studies have evidenced the protective effect of Gaba against acute kidney injury (Figure 8). According to Kim et al. (2004), the physiological changes caused by acute renal failure such as body weight and kidney weight gain, urea nitrogen and creatinine elevation, creatinine clearance reduction, sodium FE(Na) secretion, and urine osmolarity decrease in rats were significantly improved by oral administration of Gaba [117]. Moreover, the status of serum albumin decrease, urinary protein increase, and serum lipid profile was completely improved by Gaba. In addition, Gaba alleviated nephrectomy-induced oxidative stress by increasing superoxide dismutase and catalase, and decreasing lipid peroxidation in rats [118]. Furthermore, Gaba reduced tubular fibrosis, tubular atrophy, and the transforming growth factor-beta1 and fibronectin expression [119]. The acute tubular necrosis was also apparently reduced to normal proximal condition by Gaba treatment [120]. In another study, Talebi and colleagues have shown the protective effect of Gaba on kidney injury induced by renal ischemia-reperfusion in ovariectomized rats via decreasing serum levels of creatinine and blood urea nitrogen, kidney weight, and kidney tissue damage [121]. Meanwhile, the increases in alanine amino transferase and aspartate amino transferase activities, urea and creatinine levels, malondialdehyde and advanced oxidation protein levels, and oxidative damage to the kidney tissues induced by γ-irradiated- and streptozotocin-treated rats were markedly attenuated by Gaba administration in rats [122]. Especially, Gaba was observed to ameliorate kidney injury induced by renal ischemia/reperfusion injury in a gender dependent manner [123]. These results emphasized the protective effect of Gaba against the renal damage involving in renal failure.

### 2.12. Intestinal Protective Effect

Chen and colleagues have examined the beneficial roles of Gaba on intestinal mucosa in vivo [124,125]. It was shown that heat stress-induced chicken decreased the activity of Na⁺-K⁺-ATPase, maltase, sucrase, and alkaline phosphatase enzymes in intestinal mucosa [124]. Moreover, heat stress caused the marked decline in villus length, mucosa thickness, intestinal wall thickness, and crypt depth in the duodenum and ileum [125]. However, the treatment of Gaba administration markedly increased the activity of maltase, sucrase, alkaline phosphatase, and Na⁺-K⁺-ATPase [124]. Furthermore, Gaba enhanced villus length, mucosa thickness, intestinal wall thickness, and crypt depth in the duodenum and ileum [125]. It indicated that Gaba could effectively alleviate heat stress-induced damages of the intestinal mucosa. In a further study, they investigated the effect of Gaba supplementation on the growth performance, intestinal immunity, and gut microflora of the weaned piglets [126]. Notably, Gaba supplementation improved the growth performance, inhibited proinflammatory cytokines (IL-1 and IL-18) expression, promoted anti-inflammatory cytokines (IFN-γ, IL-4, and IL-10) expression, and increased the dominant microbial populations, the community richness, and diversity of the ileal microbiota. On the other hand, Xie and colleagues also investigated the effect of Gaba on colon health in mice [127]. It was observed that the female Kunming mice administrated with Gaba at doses of 40 mg/kg/d for 14 days could increase the concentrations of acetate, propionate, butyrate, and total short chain fatty acids, and decreased pH value in colonic and cecal contents. Recently, Kubota and colleagues have revealed that Gaba attenuated ischemia reperfusion-induced alterations in intestinal immunity via increasing IgA secretion, alpha-defensin-5 expression, and superoxide dismutase activity in the rat small intestine [128]. Besides, Jiang and colleagues also showed the protective effect of Gaba against intestinal mucosal barrier injury of colitis induced by 2,4,6-trinitrobenzene sulfonic acid and alcohol [129]. These results have evidenced the physiological function of Gaba in improvement and promotion of intestinal health.

### 2.13. Other Pharmaceutical Properties

Yang et al. [130] have examined the modulatory effects of Gaba on cholesterol-metabolism-associated molecules in human monocyte-derived macrophages (HMDMs). It was found that Gaba was effective in the reduction of cholesterol ester in lipid-laden HMDMs via suppressing the expression of scavenger receptor class A, lectin-like oxidized low-density lipoprotein receptor-1, and CD36, and promoting the expression of ATP-binding cassette transporter 1, ATP-binding cassette sub-family G member 1, and scavenger receptor class B type I. Moreover, the production of TNF-α was decreased and the activation of signaling pathways (p38MAPK and NF-κB) was repressed in the presence of Gaba. The inhibitory effect of Gaba on the formation of human macrophage-derived foam cells suggests its role in the prevention of atherosclerotic lesions.

Yang et al. [131] have investigated whether Gaba ameliorate fluoride-induced a thyroid injury in vivo. The model of hypothyroidism was conducted by exposing NaF (50 mg/kg) to adult male mice for 30 days. Thereafter, thyroid hormone production, oxidative stress, thyroid function-associated genes, and side effects during therapy were measured. Interestingly, Gaba supplementation remarkedly promoted the expression of thyroid thyroglobulin, thyroid peroxidase, and sodium/iodide symporter. Moreover, it improved the thyroid redox state, the expression of thyroid function-associated genes, and liver metabolic protection. These findings indicate that Gaba has a therapeutic potential in hypothyroidism.

In regarding to the growth hormone, the oral administration of Gaba was reported to elevate the resting and post-exercise immunoreactive growth hormone and immunofunctional growth hormone concentrations in humans [132]. Moreover, the administration of Gaba is likely to increase the concentrations of plasma growth hormone and the rate of protein synthesis in the rat brain [133,134]. Recently, the role of Gaba in the enhancement of muscular hypertrophy in men after progressive resistance training was also evaluated by Sakashita and colleagues [135]. They found that the combination of Gaba and whey protein was effective in increasing whole body fat-free mass, thus enhancing exercise-induced muscle hypertrophy.

Indeed, the excessive production of free radicals and oxidants causes oxidative stress that damages cell membranes and other structures such as DNA, lipids, and proteins [136]. Particularly, the damage of cell membranes and lipoproteins by hydroxyl and peroxynitrite radicals causes lipid peroxidation and formation of cytotoxic and mutagenic agents such as malondialdehyde and conjugated diene compounds [137]. Moreover, the free radicals and oxidants can change protein structure and lose enzyme activity. Various mutations may also result from oxidants-induced DNA damages. Therefore, oxidative stress can induce a variety of chronic and degenerative diseases such as cancer, cardiovascular disease, neurological disease, pulmonary disease, rheumatoid arthritis, nephropathy, and ocular disease [138]. In this sense, antioxidants play an important role in the neutralization of free radicals, protection of the cells from toxic effects, and prevention of disease pathogenesis [139]. As a result, the antioxidant activity of Gaba may partly contribute to its biological effects such as anti-hypertension, anti-diabetes, anti-cancer, antioxidant, anti-inflammation, anti-microbial, anti-allergy, hepato-protection, reno-protection, and intestinal protection.

## 3. Conclusions

The fact that consumers have paid much attention to natural products in order to promote and maintain their health. Simultaneously, various functional foods derived from natural products have been developed along with the tendency of consumers. Herein, Gaba has been evidenced as a powerful bioactive compound with numerous health beneficial effects. Thus, the functional foods produced from Gaba are believed to be able to prevent and/or treat different diseases, especially hypertension, diabetes, and neurological disorders. Whereby, the researches into large-scale production, biotechnological techniques, and high Gaba-producing strains will be remarkedly increased in food industry. However, the further testing and validation due to the safety and efficacy of Gaba consumption are necessary in clinical trials.

## Figures and Tables

**Table 1:** Neuroprotective effect of Gaba.

STT	Source	Dose, Model	Time of Treatment/Administration	Effect	Ref.
1	Kimchi-derived *Lactobacillus buchneri*	100 µg/mL, neuronal cells	24 h	Preventing neurotoxic-induced cell death	[13]
2	*Lactobacillus plantarum*-fermented chickpea milk	537.23 mg/L, PC12 cells	30 min	Preventing MnCl_2_-induced injury	[14]
3	Gaba receptor agonist	Muscimol (1 mg/kg) and baclofen (20 mg/kg), rat	30 min	Preventing brain ischemic injury and decreasing apoptosis	[15,17]
4	Gaba receptor agonist	Baclofen (10 mL/kg), rat	Once daily/five weeks	Alleviating neuronal damage and suppressing cytodestructive autophagy	[16]

**Table 2:** Neurological disorder prevention of Gaba.

STT	Source	Dose/Model	Time of Treatment/Administration	Effect	Ref.
1	Gaba-enriched rice germ	26.4 mg/3 times/day, patient	N/A	Improving sleeplessness, somnipathy, and depression	[19]
2	Gaba-rich Monascus-fermented product	2.6 mg/kg, rat	30 days	Preventing depression	[20]
3	Gaba powder from natural fermentation using lactic acid bacteria	100 mg Gaba/day, Japanese volunteers	1 week	Prevention of sleep disorder	[21]
4	Gaba (90.8%) and l-theanine (99.3%) was supplied by Neo Cremar Co. Ltd. (Seoul, Korea) and BTC Co. Ltd. (Ansan, Korea), respectively	Gaba/L-theanine mixture (100/20 mg/kg)/day, mice and rat	9 days	Decreasing sleep latency and increasing sleep duration	[22]
5	Gaba from natural fermentation using lactic acid bacteria (Pharma-GABA, Pharma Foods International Co., Japan)	Gaba/L-theanine mixture (100/200 mg/kg)/day Japanese volunteers	7 days	Increasing relaxation, diminishing anxiety, and enhancing immunity	[23]
6	Gaba-enriched product fermented by kimchi-derived lactic acid bacteria	46.69 mg/mL Gaba, mice and PC-12 cells	24 h	Improving long-term memory loss and increasing neuronal cell proliferation	[24]
7	Gaba-enriched fermented *Laminaria japonica* product	1.5 g/day, volunteers	6 weeks	Preventing cognitive impairment in the elderly	[25]

**Table 3:** Anti-hypertensive effect of Gaba.

STT	Source	Dose/Model	Time of Treatment/Administration	Effect	Ref.
1	Milk fermented by *Lactococcus lactis* DIBCA2 and *Lactobacillus plantarum* PU11	0.70 mg/ml	5 min	Inhibiting 50% ACE activity	[30]
2	Gaba form LAB-fermented soybean	1.3 mg Gaba/g soybean	10 min	Inhibiting 43% ACE activity	[33]
3	Gaba from the fermented lentils	10.42 mg Gaba/g extract	60 min	Inhibiting 92% ACE activity	[34]
4	Gaba from Wako Pure Chemicals (Tokyo)	0.3 to 300 mg Gaba/kg, rat	Every 20 min for i.v. administration	Decreasing blood pressure	[35]
5	Gaba from skim cows’ milk fermented with *Lactobacillus casei* strain Shirota and *Lactococcus lactis* YIT 2027	5 mL (102 mg Gaba/kg) of the fermented solution/kg body weight, rat	10 h	Lowering blood pressure	[36]
6	Gaba-enriched rice grains	0.1 mg–0.5 mg Gaba/kg, rat	6 weeks	Decreasing blood pressure	[38]
7	Gaba-enriched white rice	150 g of Gaba-enriched white rice (11.2 mg Gaba/100 g rice), volunteers	8 weeks	Decreasing blood pressure	[41]
8	Gaba-enriched Chingshey purple sweet potato-fermented milk by lactic acid bacteria	2.5-mL dose of fermented-milk, rat	8 weeks	Reducing both systolic blood pressure and diastolic blood pressure	[42]
9	Gaba from probiotic-fermented purple sweet potato yogurt	1500 µg/2.5 mL/kg, rat	8 weeks	Alleviating cardiac hypertrophy	[43]
10	Gaba-rich tomato	2–10 g/kg, rat	2–24 h	Decreasing blood pressure	[46]
11	Gaba-rich bread	120 g/day, patient	3 days	Decreasing blood pressure	[47]

**Table 4:** Anti-diabetic effect of Gaba.

STT	Source	Dose/Model	Time of Treatment/Administration	Effect	Ref.
1	Gaba (Source: N/A)	Dose: N/A, mice	8–15 weeks	Activating PI3-K/Akt-dependent growth and survival pathways and restoring the β-cell mass	[51]
2	Gaba (MilliporeSigma, Burlington, MA, USA)	Gaba (6 mg/mL/day), mice	10 weeks	Up-regulating β-cell proliferation and rising a distinct subpopulation of β cells	[52]
3	Gaba (Sigma, St. Louis, USA)	Gaba (2 mg/mL/day), mice	20 weeks	Reducing the concentrations of fasting blood glucose, improving glucose tolerance and insulin sensitivity, and inhibiting the body weight gain	[55]
4	Gaba from pre-germinated brown rice	Pre-germinated brown rice (1387–1546 g/day), rat	7 weeks	Decreasing blood glucose, adipocytokine PAI-1 concentration, and plasma lipid peroxide	[59]
5	Gaba from germinated brown rice	Gaba (200 mg/kg/day), rat offspring	8 weeks	Increasing adiponectin levels and reducing insulin resistance and oxidative stress	[61]
6	Gaba from blackish purple pigmented rice with a giant embryo	Diet supplemented with either 20% (*w*/*w*) germinated Keunnunjami rice powder, rat	8 weeks	Decreasing blood glucose and plasma insulin levels, adipokine concentrations, and hepatic glucose-regulating enzyme activities	[62]
7	Gaba-enriched wheat bran	15% Gaba-enriched bran, rat	8 weeks	Improving glucose homeostasis	[63]
8	Gaba from pre-germinated brown rice	The test sample contained 50 g of available carbohydrate per day for each volunteer (185 g of pre-germinated brown rice), volunteers	7 weeks	Lowering postprandial blood glucose concentration without insulin secretion increase	[65]
9	Gaba from pre-germinated brown rice	180 g of the cooked rice/three times per day, patient	14 weeks	Decreasing blood glucose and hypercholesterolemia	[66]
10	Fermented tea product	50 mg/kg, rat	120 min	Decreasing blood glucose level	[68]
11	Mung bean fermented by *Rhizopus* sp.	200 mg/kg and 1000 mg/kg, mice	240 min	Reducing blood glucose, HbA1c, cholesterol, triglyceride, and low-density lipoprotein levels	[70]
12	Yogurt fermented by *Streptococcus salivarius* subsp.	Gaba orally at a dose of 2 g/L or 4 g/L	6 weeks	Reducing blood glucose, HbA1c, cholesterol, triglyceride, and low-density lipoprotein levels	[71]
13	Soybean extract fermented by *Bacillus subtilis* MORI	500 mg/kg, mice	8 weeks	Reducing blood glucose, HbA1c, cholesterol, triglyceride, and low-density lipoprotein levels	[72]
14	Milk fermented by strain YF-L812 (*S. thermophilus*, *L. delbrueckii* subsp. *bulgaricus*), standard strains. *B. breve* KCTC 3419, and *L. sakei* LJ011. FM	Fermented milk 0.2% and 0.6%/kg/day, mice	6 weeks	Decreasing fasting blood glucose, serum insulin, insulin tolerance, total cholesterol, triglycerides, and LDL cholesterol	[73]

**Table 5:** Anti-cancer effect of Gaba.

STT	Source	Dose/Model	Time of Treatment/Administration	Effect	Ref.
1	Gaba-enriched brown rice extract	20 µL extract/well (1 × 10^5^ cells/200 mL/well), leukemia cells and HeLa cells	48 h	Retarding the proliferation rates of leukemia cells and enhancing apoptosis of leukemia cells	[77]
2	Gaba from Sigma Company (St. Louis, MO, USA)	Gaba (1–1000 µmol/L), cholangiocarcinoma QBC939 cells	24 h	Inhibiting the activity and expression of MMP-2 and MMP-9	[79]
3	Gaba was purchased from Sigma-Aldrich, Shanghai, China	Gaba (100 µM), Colon cancer cells	72 h	Inhibiting on cell proliferation and metastasis	[80]
4	Gaba from Sigma Company (St. Louis, MO, USA)	Gaba (1000 mg/kg), rat	25 weeks	Decreasing the number of gastric cancers of the glandular stomach	[82]
5	Gaba from Sigma Company (St. Louis, MO, USA)	Gaba (10 µM), Human liver cancer cells	24 h	Reducing intrahepatic liver metastasis and inhibiting human liver cancer cell migration and invasion	[83]

## References

[1] Olsen R.W., Betz H., Siegel G.J., Albers R.W., Brady S.T., Price D.L. (2006). GABA and glycine. Basic Neurochemistry.

[2] Martin D.L., Olsen R.W. (2000). GABA in the Nervous System: The View at 50 Years.

[3] Madsen K.K., Clausen R.P., Larsson O.M., Krogsgaard-Larsen P., Schousboe A., White H.S. (2009). Synaptic and extrasynaptic GABA transporters as targets for anti-epileptic drugs. J. Neurochem..

[4] Mann E.O., Kohl M.M., Paulsen O. (2009). Distinct roles of GABA(A) and GABA(B) receptors in balancing and terminating persistent cortical activity. J. Neurosci..

[5] Stagg C.J., Bachtiar V., Johansen-Berg H. (2011). The role of GABA in human motor learning. Curr. Biol..

[6] Nuss P. (2015). Anxiety disorders and GABA neurotransmission: A disturbance of modulation. Neuropsychiatr. Dis. Treat..

[7] Wagner S., Castel M., Gainer H., Yarom Y. (1997). GABA in the mammalian suprachiasmatic nucleus and its role in diurnal rhythmicity. Nature.

[8] Gottesmann C. (2002). GABA mechanisms and sleep. Neuroscience.

[9] Plante D.T., Jensen J.E., Schoerning L., Winkelman J.W. (2012). Reduced γ-aminobutyric acid in occipital and anterior cingulate cortices in primary insomnia: A link to major depressive disorder?. Neuropsychopharmacology.

[10] Rashmi D., Zanan R., John S., Khandagale K., Nadaf A., Rahman A. (2018). γ-aminobutyric acid (GABA): Biosynthesis, role, commercial production, and application. Study in Natural Products Chemistry.

[11] Kaminsky N., Bihari O., Kanner S., Barzilai A. (2016). Connecting malfunctioning glial cells and brain degenerative disorders. Genom. Proteom. Bioinform..

[12] Bagli E., Goussia A., Moschos M.M., Agnantis N., Kitsos G. (2016). Natural compounds and neuroprotection: Mechanisms of action and novel delivery systems. In Vivo.

[13] Cho Y.R., Chang J.Y., Chang H.C. (2007). Production of gamma-aminobutyric acid (GABA) by *Lactobacillus buchneri* isolated from kimchi and its neuroprotective effect on neuronal cells. J. Microbiol. Biotechnol..

[14] Li W., Wei M., Wu J., Rui X., Dong M. (2016). Novel fermented chickpea milk with enhanced level of γ-aminobutyric acid and neuroprotective effect on PC12 cells. Peer J.

[15] Zhou C., Li C., Yu H.M., Zhang F., Han D., Zhang G.Y. (2008). Neuroprotection of gamma-aminobutyric acid receptor agonists via enhancing neuronal nitric oxide synthase (Ser847) phosphorylation through increased neuronal nitric oxide synthase and PSD95 interaction and inhibited protein phosphatase activity in cerebral ischemia. J. Neurosci. Res..

[16] Liu L., Li C.J., Lu Y., Zong X.G., Luo C., Sun J., Guo L.J. (2015). Baclofen mediates neuroprotection on hippocampal CA1 pyramidal cells through the regulation of autophagy under chronic cerebral hypoperfusion. Sci. Rep..

[17] Wei X.W., Yan H., Xu B., Wu Y.P., Li C., Zhang G.Y. (2012). Neuroprotection of co-activation of GABA receptors by preventing caspase-3 denitrosylation in KA-induced seizures. Brain. Res. Bull..

[18] Parvez M.K. (2018). Natural or plant products for the treatment of neurological disorders: Current knowledge. Curr. Drug. Metab..

[19] Okada T., Sugishita T., Murakami T., Murai H., Saikusa T., Horino T., Onoda A., Kajimoto O., Takahashi R., Takahashi T. (2000). Effect of the defatted rice germ enriched with GABA for sleeplessness, depression, autonomic disorder by oral administration. J. Jpn. Soc. Food Sci..

[20] Chuang C.Y., Shi Y.C., You H.P., Lo Y.H., Pan T.M. (2011). Antidepressant effect of GABA-rich monascus-fermented product on forced swimming rat model. J. Agric. Food Chem..

[21] Yamatsu A., Yamashita Y., Pandharipande T., Maru I., Kim M. (2016). Effect of oral γ-aminobutyric acid (GABA) administration on sleep and its absorption in humans. Food Sci. Biotechnol..

[22] Kim S., Jo K., Hong K.B., Han S.H., Suh H.J. (2019). GABA and l-theanine mixture decreases sleep latency and improves NREM sleep. Pharm. Biol..

[23] Abdou A.M., Higashiguchi S., Horie K., Kim M., Hatta H., Yokogoshi H. (2006). Relaxation and immunity enhancement effect of gamma-aminobutyric acid (GABA) administration in humans. BioFactors.

[24] Seo Y.C., Choi W.Y., Kim J.S., Lee C.G., Ahn J.H., Cho H.Y., Lee S.H., Cho J.S., Joo S.J., Lee H.Y. (2012). Enhancement of the cognitive effects of GABA from monosodium glutamate fermentation by *Lactobacillus sakei* B2-16. Food Biotechnol..

[25] Reid S.N.S., Ryu J.K., Kim Y., Jeon B.H. (2018). The effects of fermented *Laminaria japonica* on short-term working memory and physical fitness in the elderly. Evid. Based Complement. Altern. Med..

[26] Reid S.N.S., Ryu J.K., Kim Y.S., Jeon B.H. (2018). GABA-enriched fermented *Laminaria japonica* improves cognitive impairment and neuroplasticity in scopolamine- and ethanol-induced dementia model mice. Nutr. Res. Pract..

[27] Choi W.C., Reid S.N.S., Ryu J.K., Kim Y., Jo Y.H., Jeon B.H. (2016). Effects of γ-aminobutyric acid-enriched fermented sea tangle (*Laminaria japonica*) on brain derived neurotrophic factor-related muscle growth and lipolysis in middle aged women. Algae.

[28] Schellack N., Naicker P. (2015). Hypertension: A review of antihypertensive medication, past and present. S. Afr. Pharm. J..

[29] Murray B.A., FitzGerald R.J. (2007). Angiotensin converting enzyme inhibitory peptides derived from food proteins: Biochemistry, bioactivity and production. Curr. Pharm. Des..

[30] Nejati F., Rizzello C.G., Cagno R.D., Sheikh-Zeinoddin M., Diviccaro A., Minervini F., Gobbetti M. (2013). Manufacture of a functional fermented milk enriched of angiotensin-I converting enzyme (ACE)-inhibitory peptides and γ-amino butyric acid (GABA). Food Sci. Technol..

[31] Tung Y.T., Lee B.H., Liu C.F., Pan T.M. (2011). Optimization of culture condition for ACEI and GABA production by lactic acid bacteria. J. Food Sci..

[32] Jang E.K., Kim N.Y., Ahn H.J., Ji G.E. (2015). γ-Aminobutyric acid (GABA) production and angiotensin-I converting enzyme (ACE) inhibitory activity of fermented soybean containing sea tangle by the co-culture of *Lactobacillus brevis* with *Aspergillus oryzae*. J. Microbiol. Biotechnol..

[33] Sang V.T., Uyen L.P., Hung N.D. (2018). The increased gamma-aminobutyric acid content by optimizing fermentation conditions of bacteria from kimchi and investigation of its biological activities. EurAsian J. Biosci..

[34] Torino M.I., Limón R.I., Martínez-Villaluenga C., Mäkinen S., Pihlanto A., Vidal-Valverde C., Frias J. (2013). Antioxidant and antihypertensive properties of liquid and solid state fermented lentils. Food Chem..

[35] Kimura M., Hayakawa K., Sansawa H. (2002). Involvement of gamma-aminobutyric acid (GABA) B receptors in the hypotensive effect of systemically administered GABA in spontaneously hypertensive rats. Jpn. J. Pharmacol..

[36] Hayakawa K., Kimura M., Kasaha K., Matsumoto K., Sansawa H., Yamori Y. (2004). Effect of a gamma-aminobutyric acid-enriched dairy product on the blood pressure of spontaneously hypertensive and normotensive Wistar-Kyoto rats. Br. J. Nutr..

[37] Matsubara F., Ueno H., Kentaro T., Tadano K., Suyama T., Imaizumi K., Suzuki T., Magata K., Kikuchi N. (2002). Effects of GABA supplementation on blood pressure and safety in adults with mild hypertension. Jpn. Pharmacol. Ther..

[38] Akama K., Kanetou J., Shimosaki S., Kawakami K., Tsuchikura S., Takaiwa F. (2009). Seed-specific expression of truncated OsGAD2 produces GABA-enriched rice grains that influence a decrease in blood pressure in spontaneously hypertensive rats. Transgenic Res..

[39] Ebizuka H., Ihara M., Arita M. (2009). Antihypertensive effect of pre-germinated brown rice in spontaneously hypertensive rats. Food Sci. Technol. Res..

[40] Kawakami K., Yamada K., Yamada T., Nabika T., Nomura M. (2018). Antihypertensive effect of γ-aminobutyric acid-enriched brown rice on spontaneously hypertensive rats. J. Nutr. Sci. Vitaminol. (Tokyo).

[41] Nishimura M., Yoshida S., Haramoto M., Mizuno H., Fukuda T., Kagami-Katsuyama H., Tanaka A., Ohkawara T., Sato Y., Nishihira J. (2016). Effects of white rice containing enriched gamma-aminobutyric acid on blood pressure. J. Tradit. Complement. Med..

[42] Tsai C.C., Chiu T.H., Ho C.Y., Lin P.P., Wu T.Y. (2013). Effects of anti-hypertension and intestinal microflora of spontaneously hypertensive rats fed gammaaminobutyric acid-enriched Chingshey purple sweet potato fermented milk by lactic acid bacteria. Afr. J. Microbiol. Res..

[43] Lin P.P., Hsieh Y.M., Kuo W.W., Lin C.C., Tsai F.J., Tsai C.H., Huang C.Y., Tsai C.C. (2012). Inhibition of cardiac hypertrophy by probiotic-fermented purple sweet potato yogurt in spontaneously hypertensive rat hearts. Int. J. Mol. Med..

[44] Suwanmanon K., Hsieh P.C. (2014). Effect of γ-aminobutyric acid and nattokinase-enriched fermented beans on the blood pressure of spontaneously hypertensive and normotensive Wistar-Kyoto rats. J. Food Drug. Anal..

[45] Aoki H., Furuya Y., Endo Y., Fujimoto K. (2003). Effect of gamma-aminobutyric acid-enriched tempeh-like fermented soybean (GABA-Tempeh) on the blood pressure of spontaneously hypertensive rats. Biosci. Biotechnol. Biochem..

[46] Yoshimura M., Toyoshi T., Sano A., Izumi T., Fujii T., Konishi C., Inai S., Matsukura C., Fukuda N., Ezura H. (2010). Antihypertensive effect of a gamma-aminobutyric acid rich tomato cultivar ‘DG03-9’ in spontaneously hypertensive rats. J. Agric. Food Chem..

[47] Becerra-Tomás N., Guasch-Ferré M., Quilez J., Merino J., Ferré R., Díaz-López A., Bulló M., Hernández-Alonso P., Palau-Galindo A., Salas-Salvadó J. (2015). Effect of functional bread rich in potassium, γ-aminobutyric acid and angiotensin-converting enzyme inhibitors on blood pressure, glucose metabolism and endothelial function: A double-blind randomized crossover clinical trial. Medicine (Baltimore).

[48] Kharroubi A.T., Darwish H.M. (2015). Diabetes mellitus: The epidemic of the century. World J. Diabetes.

[49] Li G., Zhang P., Wang J., Gregg E.W., Yang W., Gong Q. (2008). The long-term effect of life style interventions to prevent diabetes in the China Da Qing diabetes prevention study: A 20-year follow-up study. Lancet.

[50] Babiker A., Dubayee M.A. (2017). Anti-diabetic medications: How to make a choice?. Sudan J. Paediatr..

[51] Soltani N., Qiu H., Aleksic M., Glinka Y., Zhao F., Liu R., Li Y., Zhang N., Chakrabarti R., Ng T. (2011). GABA exerts protective and regenerative effects on islet beta cells and reverses diabetes. Proc. Natl. Acad. Sci. USA.

[52] Untereiner A., Abdo S., Bhattacharjee A., Gohil H., Pourasgari F., Ibeh N., Lai M., Batchuluun B., Wong A., Khuu N. (2019). GABA promotes β-cell proliferation, but does not overcome impaired glucose homeostasis associated with diet-induced obesity. FASEB J..

[53] Liu W., Son D.O., Lau H.K., Zhou Y., Prud’homme G.J., Jin T., Wang Q. (2017). Combined oral administration of GABA and DPP-4 inhibitor prevents beta cell damage and promotes beta cell regeneration in mice. Front. Pharmacol..

[54] Bansal P., Wang S., Liu S., Xiang Y.Y., Lu W.Y., Wang Q. (2011). GABA coordinates with insulin in regulating secretory function in pancreatic INS-1 β-cells. PLoS ONE.

[55] Tian J., Dang H.N., Yong J., Chui W.S., Dizon M.P., Yaw C.K., Kaufman D.L. (2011). Oral treatment with γ-aminobutyric acid improves glucose tolerance and insulin sensitivity by inhibiting inflammation in high fat diet-fed mice. PLoS ONE.

[56] Huang C.Y., Kuo W.W., Wang H.F., Lin C.J., Lin Y.M., Chen J.L., Kuo C.H., Chen P.K., Lin J.Y. (2014). GABA tea ameliorates cerebral cortex apoptosis and autophagy in streptozotocin-induced diabetic rats. J. Funct. Foods.

[57] Imam M.U., Azmi N.H., Bhanger M.I., Ismail N., Ismail M. (2012). Antidiabetic properties of germinated brown rice: A systematic review. J. Evid. Based Complement. Altern. Med..

[58] Sivamaruthi B.S., Kesika P., Prasanth M.I., Chaiyasut C. (2018). A mini review on antidiabetic properties of fermented foods. Nutrients.

[59] Hagiwara H., Seki T., Ariga T. (2004). The effect of pre-germinated brown rice intake on blood glucose and PAI-1 levels in streptozotocin-induced diabetic rats. Biosci. Biotechnol. Biochem..

[60] Torimitsu M., Nagase R., Yanagi M., Homma M., Sasai Y., Ito Y., Hayamizu K., Nonaka S., Hosono T., Kise M. (2010). Replacing white rice with pre-germinated brown rice mildly ameliorates hyperglycemia and imbalance of adipocytokine levels in type 2 diabetes model rats. J. Nutr. Sci. Vitaminol. (Tokyo).

[61] Adamu H.A., Imam M.U., Ooi D.J., Esa N.M., Rosli R., Ismail M. (2016). Perinatal exposure to germinated brown rice and its gamma amino-butyric acid-rich extract prevents high fat diet-induced insulin resistance in first generation rat offspring. Food Nutr. Res..

[62] Chung S.I., Jin X., Kang M.Y. (2019). Enhancement of glucose and bone metabolism in ovariectomized rats fed with germinated pigmented rice with giant embryo (*Oryza sativa* L. cv. Keunnunjami). Food Nutr. Res..

[63] Shang W., Si X., Zhou Z., Strappe P., Blanchard C. (2018). Wheat bran with enriched gamma-aminobutyric acid attenuates glucose intolerance and hyperinsulinemia induced by a high-fat diet. Food Funct..

[64] Si X., Shang W., Zhou Z., Shui G., Lam S.M., Blanchard C., Strappe P. (2018). Gamma-aminobutyric acid enriched rice bran diet attenuates insulin resistance and balances energy expenditure via modification of gut microbiota and short-chain fatty acids. J. Agric. Food Chem..

[65] Ito Y., Mizukuchi A., Kise M., Aoto H., Yamamoto S., Yoshihara R., Yokoyama J. (2005). Postprandial blood glucose and insulin responses to pre-germinated brown rice in healthy subjects. J. Med. Investig..

[66] Hsu T.F., Kise M., Wang M.F., Ito Y., Yang M.D., Aoto H., Yoshihara R., Yokoyama J., Kunii D., Yamamoto S. (2008). Effects of pre-germinated brown rice on blood glucose and lipid levels in free-living patients with impaired fasting glucose or type 2 diabetes. J. Nutr. Sci. Vitaminol. (Tokyo).

[67] Hayakawa T., Suzuki S., Kobayashi S., Fukutomi T., Ide M., Ohno T., Ohkouchi M., Taki M., Miyamoto T., Nimura T. (2009). Effect of pre-germinated brown rice on metabolism of glucose and lipid in patients with diabetes mellitus type 2. J. Jpn. Assoc. Rural Med..

[68] Tamaya K., Matsui T., Toshima A., Noguchi M., Ju Q., Miyata Y., Tanaka T., Tanaka K. (2010). Suppression of blood glucose level by a new fermented tea obtained by tea-rolling processing of loquat (*Eriobotrya japonica*) and green tea leaves in disaccharide-loaded Sprague-Dawley rats. J. Sci. Food Agric..

[69] Lee S.Y., Park S.L., Nam Y.D., Lee S.H. (2013). Anti-diabetic effects of fermented green tea in KK-Ay diabetic mice. Korean J. Food Sci. Technol..

[70] Yeap S.K., Ali N.M., Yusof H.M., Alitheen N.B., Beh B.K., Ho W.Y., Koh S.P., Long K. (2012). Antihyperglycemic effects of fermented and nonfermented mung bean extracts on alloxan-induced-diabetic mice. J. Biomed. Biotechnol..

[71] Chen L., Zhao H., Zhang C., Lu Y., Zhu X., Lu Z. (2016). γ-Aminobutyric acid-rich yogurt fermented by *Streptococcus salivarius* subsp. thermophiles fmb5 apprars to have anti-diabetic effect on streptozotocin-induced diabetic mice. J. Funct. Foods.

[72] Nam H., Jung H., Karuppasamy S., Park Y.S., Cho Y.S., Lee J.Y., Seong S.I., Suh J.G. (2012). Anti-diabetic effect of the soybean extract fermented by *Bacillus subtilis* MORI in db/db mice. Food Sci. Biotechnol..

[73] Song K., Song I.B., Gu H.J., Na J., Kim S., Yang H.S., Lee S.C., Huh C., Kwon J. (2016). Anti-diabetic effect of fermented milk containing conjugated linoleic acid on type II diabetes mellitus. Korean J. Food Sci. Anim. Resour..

[74] Ostadrahimi A., Taghizadeh A., Mobasseri M., Farrin N., Payahoo L., Gheshlaghi Z.B., Vahedjabbari M. (2015). Effect of probiotic fermented milk (kefir) on glycemic control and lipid profile in type 2 diabetic patients: A randomized double-blind placebo-controlled clinical trial. Iran. J. Public Health.

[75] Alihosseini N., Moahboob S.A., Farrin N., Mobasseri M., Taghizadeh A., Ostadrahimi A.R. (2017). Effect of probiotic fermented milk (kefir) on serum level of insulin and homocysteine in type 2 diabetes patients. Acta Endocrinol..

[76] Ribas V., García-Ruiz C., Fernández-Checa J.C. (2016). Mitochondria, cholesterol and cancer cell metabolism. Clin. Transl. Med..

[77] Oh C.H., Oh S.H. (2004). Effects of germinated brown rice extracts with enhanced levels of GABA on cancer cell proliferation and apoptosis. J. Med. Food.

[78] Schuller H.M., Al-Wadei H.A., Majidi M. (2008). Gamma-aminobutyric acid, a potential tumor suppressor for small airway-derived lung adenocarcinoma. Carcinogenesis.

[79] Huang Q., Liu C., Wang C., Hu Y., Qiu L., Xu P. (2011). Neurotransmitter γ-aminobutyric acid-mediated inhibition of the invasive ability of cholangiocarcinoma cells. Oncol. Lett..

[80] Song L., Du A., Xiong Y., Jiang J., Zhang Y., Tian Z., Yan H. (2016). γ-Aminobutyric acid inhibits the proliferation and increases oxaliplatin sensitivity in human colon cancer cells. Tumour. Biol..

[81] Al-Wadei H.A., Al-Wadei M.H., Ullah M.F., Schuller H.M. (2012). Celecoxib and GABA cooperatively prevent the progression of pancreatic cancer in vitro and in xenograft models of stress-free and stress-exposed mice. PLoS ONE.

[82] Tatsuta M., Iishi H., Baba M., Nakaizumi A., Ichii M., Taniguchi H. (1990). Inhibition by gamma-amino-n-butyric acid and baclofen of gastric carcinogenesis induced by N-methyl-N’-nitro-N-nitrosoguanidine in Wistar rats. Cancer Res..

[83] Chen Z.A., Bao M.Y., Xu Y.F., Zha R.P., Shi H.B., Chen T.Y., He X.H. (2012). Suppression of human liver cancer cell migration and invasion via the GABAA receptor. Cancer Biol. Med..

[84] Zhang M., Gong Y., Assy N., Minuk Y. (2000). Increased GABAergic activity inhibits a-fetoprotein mRNA expression and the proliferation activity of the HepG2 human hepatocellular carcinoma cell line. J. Hepatol..

[85] Phaniendra A., Jestadi D.B., Periyasamy L. (2015). Free radicals: Properties, sources, targets, and their implication in various diseases. Indian J. Clin. Biochem..

[86] Lobo V., Patil A., Phatak A., Chandra N. (2010). Free radicals, antioxidants and functional foods: Impact on human health. Pharmacogn. Rev..

[87] Deng Y., Xu L., Zeng X., Li Z., Qin B., He N. (2010). New perspective of GABA as an inhibitor of formation of advanced lipoxidation end-products: it’s interaction with malondiadehyde. J. Biomed. Nanotechnol..

[88] Deng Y., Wang W., Yu P., Xi Z., Xu L., Li X., He N. (2013). Comparison of taurine, GABA, Glu, and Asp as scavengers of malondialdehyde in vitro and in vivo. Nanoscale Res. Lett..

[89] Tang X., Yu R., Zhou Q., Jiang S., Le G. (2018). Protective effects of γ-aminobutyric acid against H_2_O_2_-induced oxidative stress in RIN-m5F pancreatic cells. Nutr. Metab. (Lond.).

[90] Zhu Z., Shi Z., Xie C., Gong W., Hu Z., Peng Y. (2019). A novel mechanism of gamma-aminobutyric acid (GABA) protecting human umbilical vein endothelial cells (HUVECs) against H_2_O_2_-induced oxidative injury. Comp. Biochem. Physiol. C Toxicol. Pharmacol..

[91] El-Hady A.M.A., Gewefel H.S., Badawi M.A., Eltahawy N.A. (2017). Gamma-aminobutyric acid ameliorates gamma rays-induced oxidative stress in the small intestine of rats. J. Basic Appl. Zool..

[92] Eltahawy N.A., Saada H.N., Hammad A.S. (2017). Gamma amino butyric acid attenuates brain oxidative damage associated with insulin alteration in streptozotocin-treated rats. Indian J. Clin. Biochem..

[93] Lee B.J., Kim J.S., Kang Y.M., Lim J.H., Kim Y.M., Lee M.S., Jeong M.H., Ahn C.B., Je J.Y. (2010). Antioxidant activity and γ-aminobutyric acid (GABA) content in sea tangle fermented by *Lactobacillus brevis* BJ20 isolated from traditional fermented foods. Food Chem..

[94] Md Zamri N.D., Imam M.U., Abd Ghafar S.A., Ismail M. (2014). Antioxidative effects of germinated brown rice-derived extracts on H_2_O_2_-induced oxidative stress in HepG2 cells. Evid. Based Complement. Altern. Med..

[95] Phuapaiboon P. (2017). Gamma-aminobutyric acid, total anthocyanin content and antioxidant activity of vinegar brewed from germinated pigmented rice. Pakistan J. Nutr..

[96] Chen L., Deng H., Cui H., Fang J., Zuo Z., Deng J., Li Y., Wang X., Zhao L. (2017). Inflammatory responses and inflammation-associated diseases in organs. Oncotarget.

[97] Abdulkhaleq L.A., Assi M.A., Abdullah R., Zamri-Saad M., Taufiq-Yap Y.H., Hezmee M. (2018). The crucial roles of inflammatory mediators in inflammation: A review. Vet. World.

[98] Han D., Kim H.Y., Lee H.J., Shim I., Hahm D.H. (2007). Wound healing activity of gamma-aminobutyric acid (GABA) in rats. J. Microbiol. Biotechnol..

[99] Prud’homme G., Glinka Y., Wang Q. (2013). GABA exerts anti-inflammatory and immunosuppressive effects (P5175). J. Immunol..

[100] Choi J.I., Yun I.H., Jung Y.J., Lee E.H., Nam T.J., Kim Y.M. (2012). Effects of γ-aminobutyric acid (gaba)-enriched sea tangle *Laminaria japonica* extract on lipopolysaccharide-induced inflammation in mouse macrophage (RAW 264.7) cells. Fish Aquat. Sci..

[101] Tuntipopipat S., Muangnoi C., Thiyajai P., Srichamnong W., Charoenkiatkul S., Praengam K. (2015). A bioaccessible fraction of parboiled germinated brown rice exhibits a higher anti-inflammatory activity than that of brown rice. Food Funct..

[102] Scandolera A., Hubert J., Humeau A., Lambert C., De Bizemont A., Winkel C., Kaouas A., Renault J.H., Nuzillard J.M., Reynaud R. (2018). GABA and GABA-alanine from the red microalgae *Rhodosorus marinus* exhibit a significant neuro-soothing activity through inhibition of neuro-inflammation mediators and positive regulation of TRPV1-related skin sensitization. Mar. Drugs.

[103] Sokovic Bajic S., Djokic J., Dinic M., Veljovic K., Golic N., Mihajlovic S., Tolinacki M. (2019). GABA-producing natural dairy isolate from artisanal zlatar cheese attenuates gut inflammation and strengthens gut epithelial barrier in vitro. Front. Microbiol..

[104] Mau J.L., Chiou S.Y., Hsu C.A., Tsai H.L., Lin S.D. (2012). Antimutagenic and antimicrobial activities of γ-aminobutyric acid (Gaba) tea extract. Int. Conf. Nutr. Food Sci..

[105] Dagorn A., Hillion M., Chapalain A., Lesouhaitier O., Duclairoir Poc C., Vieillard J., Chevalier S., Taupin L., Le Derf F., Feuilloley M.G. (2013). Gamma-aminobutyric acid acts as a specific virulence regulator in *Pseudomonas aeruginosa*. Microbiology.

[106] Kim J.K., Kim Y.S., Lee H.M., Jin H.S., Neupane C., Kim S., Lee S.H., Min J.J., Sasai M., Jeong J.H. (2018). GABAergic signaling linked to autophagy enhances host protection against intracellular bacterial infections. Nat. Commun..

[107] Galli S.J., Tsai M. (2012). IgE and mast cells in allergic disease. Nat. Med..

[108] Hori A., Hara T., Honma K., Joh T. (2008). Suppressive effect of γ-aminobutyric acid (GABA) on histamine release in rat basophilic RBL-2H3 cells. Bull. Fac. Agric. Niigata Univ..

[109] Kawasaki A., Hara T., Joh T. (2014). Inhibitory effect of γ-aminobutyric acid (GABA) on histamine release from rat basophilic leukemia RBL-2H3 cells and rat peritoneal exudate cells. Nippon Shokuhin Kagaku Kogaku Kaishi.

[110] Hokazono H., Omori T., Ono K. (2010). Effects of single and combined administration of fermented barley extract and γ -aminobutyric acid on the development of atopic dermatitis in NC/Nga mice. Biosci. Biotechnol. Biochem..

[111] Nagy L.E. (2004). Molecular aspects of alcohol metabolism: Transcription factors involved in early ethanol-induced liver injury. Annu. Rev. Nutr..

[112] Oh S.H., Soh J.R., Cha Y.S. (2003). Germinated brown rice extract shows a nutraceutical effect in the recovery of chronic alcohol-related symptoms. J. Med. Food.

[113] Lee B.J., Senevirathne M., Kim J.S., Kim Y.M., Lee M.S., Jeong M.H., Kang Y.M., Kim J.I., Nam B.H., Ahn C.B. (2010). Protective effect of fermented sea tangle against ethanol and carbon tetrachloride-induced hepatic damage in Sprague-Dawley rats. Food Chem. Toxicol..

[114] Cha J.Y., Lee B.J., Je J.Y., Kang Y.M., Kim Y.M., Cho Y.S. (2011). GABA-enriched fermented *Laminaria japonica* protects against alcoholic hepatotoxicity in Sprague-Dawley rats. Fish Aquat. Sci..

[115] Kang Y.M., Qian Z.J., Lee B.J., Kim Y.M. (2011). Protective effect of GABA-enriched fermented sea tangle against ethanol-induced cytotoxicity in HepG2 Cells. Biotechnol. Bioprocess E.

[116] Makris K., Spanou L. (2016). Acute kidney injury: Definition, pathophysiology and clinical phenotypes. Clin. Biochem. Rev..

[117] Kim H.Y., Yokozawa T., Nakagawa T., Sasaki S. (2004). Protective effect of gamma-aminobutyric acid against glycerol-induced acute renal failure in rats. Food Chem. Toxicol..

[118] Sasaki S., Yokozawa T., Cho E.J., Oowada S., Kim M. (2006). Protective role of gamma-aminobutyric acid against chronic renal failure in rats. J. Pharm. Pharmacol..

[119] Sasaki S., Tohda C., Kim M., Yokozawa T. (2007). Gamma-aminobutyric acid specifically inhibits progression of tubular fibrosis and atrophy in nephrectomized rats. Biol. Pharm. Bull..

[120] Ali B.H., Al-Salam S., Al Za’abi M., Al Balushi K.A., AlMahruqi A.S., Beegam S., Al-Lawatia I., Waly M.I., Nemmar A. (2015). Renoprotective effects of gamma-aminobutyric acid on cisplatin-induced acute renal injury in rats. Basic Clin. Pharmacol. Toxicol..

[121] Talebi N., Nematbakhsh M., Monajemi R., Mazaheri S., Talebi A., Vafapour M. (2016). The protective effect of γ-aminobutyric acid on kidney injury induced by renal ischemia-reperfusion in ovariectomized estradiol-treated rats. Int. J. Prev. Med..

[122] Saada H.N., Eltahawy N.A., Hammad A.S., Morcos N.Y.S. (2016). Gamma amino butyric acid attenuates liver and kidney damage associated with insulin alteration in γ-irradiated and streptozotocin-treated rats. Arab. J. Nucl. Sci. Appl..

[123] Vafapour M., Nematbakhsh M., Monajemi R., Mazaheri S., Talebi A., Talebi N., Shirdavani S. (2015). Effect of γ-aminobutyric acid on kidney injury induced by renal ischemia-reperfusion in male and female rats: Gender-related difference. Adv. Biomed. Res..

[124] Chen Z., Xie J., Wang B., Tang J. (2014). Effect of γ-aminobutyric acid on digestive enzymes, absorption function, and immune function of intestinal mucosa in heat-stressed chicken. Poult. Sci..

[125] Chen Z., Xie J., Hu M.Y., Tang J., Shao Z.F., Li M.H. (2015). Protective effects of γ-aminobutyric acid (gaba) on the small intestinal mucosa in heat-stressed wenchang chicken. J. Anim. Plant Sci..

[126] Chen S., Tan B., Xia Y., Liao S., Wang M., Yin J., Wang J., Xiao H., Qi M., Bin P. (2019). Effects of dietary gamma-aminobutyric acid supplementation on the intestinal functions in weaning piglets. Food Funct..

[127] Xie M., Chen H.H., Nie S.P., Yin J.Y., Xie M.Y. (2017). Gamma-aminobutyric acid increases the production of short-chain fatty acids and decreases pH values in mouse colon. Molecules.

[128] Kubota A., Kobayashi M., Sarashina S., Takeno R., Yasuda G., Narumi K., Furugen A., Takahashi-Suzuki N., Iseki K. (2018). Gamma-aminobutyric acid (GABA) attenuates ischemia reperfusion-induced alterations in intestinal immunity. Biol. Pharm. Bull..

[129] Jiang T., Yue Y., Li F. (2018). Improvement of gamma-aminobutyric acid on intestinal mucosalbarrier injury of colitis induced by 2,4,6-trinitrobenzene sulfonic acid and alcohol. Herald Med..

[130] Yang Y., Lian Y.T., Huang S.Y., Yang Y., Cheng L.X., Liu K. (2014). GABA and topiramate inhibit the formation of human macrophage-derived foam cells by modulating cholesterol-metabolism-associated molecules. Cell. Physiol. Biochem..

[131] Yang H., Xing R., Liu S., Yu H., Li P. (2019). Analysis of the protective effects of γ-aminobutyric acid during fluoride-induced hypothyroidism in male Kunming mice. Pharm. Biol..

[132] Powers M.E., Borst S.E., McCoy S.C., Conway R., Yarrow J.F. (2003). The effects of gamma aminobutyric acid on growth hormone secretion at rest and following exercise. Med. Sci. Sports Exerc..

[133] Tujioka K., Okuyama S., Yokogoshi H., Fukaya Y., Hayase K., Horie K., Kim M. (2007). Dietary gamma-aminobutyric acid affects the brain protein synthesis rate in young rats. Amino Acids.

[134] Tujioka K., Ohsumi M., Horie K., Kim M., Hayase K., Yokogoshi H. (2009). Dietary gamma-aminobutyric acid affects the brain protein synthesis rate in ovariectomized female rats. J. Nutr. Sci. Vitaminol. (Tokyo).

[135] Sakashita M., Nakamura U., Horie N., Yokoyama Y., Kim M., Fujita S. (2019). Oral supplementation using gamma-aminobutyric acid and whey protein improves whole body fat-free mass in men after resistance training. J. Clin. Med. Res..

[136] Droge W. (2002). Free radicals in the physiological control of cell function. Physiol. Rev..

[137] Pacher P., Beckman J.S., Liaudet L. (2007). Nitric oxide and peroxynitrite in health and disease. Physiol. Rev..

[138] Pham-Huy L.A., He H., Pham-Huy C. (2008). Free radicals, antioxidants in disease and health. Int. J. Biomed. Sci..

[139] Willcox J.K., Ash S.L., Catignani G.L. (2004). Antioxidants and prevention of chronic disease. Crit. Rev. Food. Sci. Nutr..

